# Students’ English-medium instruction motivation in three English-medium instruction courses in China

**DOI:** 10.3389/fpsyg.2022.1077852

**Published:** 2023-01-04

**Authors:** Mengjia Zhang, Elisabet Pladevall-Ballester

**Affiliations:** ^1^Teacher Education U-G-S Collaborative Innovation Research Center, School of English Education, Xi’an International Studies University, Xi’an, China; ^2^Departament de Filologia Anglesa i de Germanística, Universitat Autònoma de Barcelona, Barcelona, Spain

**Keywords:** English-medium instruction, motivation, anxiety, development, pre-post questionnaires, focus group interviews, China

## Abstract

English-medium instruction (EMI) has been spreading rapidly as the result of China’s movement to internationalize its HEIs (higher education institutions). However, there is a dearth of research studies on students’ motivation in EMI contexts, which should not only explore students’ Foreign Language Learning (FLL) motivation in isolation but the highlights of integrating both content and language learning as a complex. This paper specifically reports on the development of students’ EMI motivation and anxiety over one semester and compares three disciplines: International Trade, Film Production, and Project Management. Pre-post questionnaires and post focus group interviews were administered to students. Results showed that students generally had high EMI motivation and anxiety though the levels decreased from pre to post phases. The International Trade group had greater motivation, particularly instrumental motivation. Findings are discussed in relation to the existing literature and the local context. Pedagogical and institutional-level implications for policies are also provided.

## 1. Introduction

As China aimed to internationalize its higher education institutions (HEIs), English-medium instruction (EMI) has been widely adopted in Chinese universities (Guo et al., 2018; Xie and Curle, 2022). English is in the position of a dominant *lingua franca* in higher education (HE), enabling the possibility of student and staff mobility as well as the exchange of academic work and research (Dafouz and Smit, 2016). EMI can be referred to as “the use of the English language to teach academic subjects other than English itself in countries or jurisdictions where the first language of the majority of the population is not English” (Macaro, 2018, p. 19).

EMI was officially implemented in Ministry of Education of the People’s Republic of China (2001) with the purpose of achieving higher quality for undergraduate education. Unlike other contexts, EMI in China is known as bilingual teaching (Shuang yu jiao xue), covering EMI-only or the bilingual EMI and Chinese-medium instruction (CMI) models. Empirical research data in China, however, is scarce, especially when compared with the vigorous growth of EMI courses/programs across the country; hence, researchers’ call to empirically evaluate EMI implementation in China continues to draw scholarly attention to investigate how students can be motivated in contexts where English is not the targeted language but used merely as the medium of instruction to deliver content knowledge (Lei and Hu, 2014; Galloway et al., 2017; Guo et al., 2018; Xie and Curle, 2022).

As Carrió-Pastor (2020) noted, EMI requires a great deal of learning motivation on the part of students to be able to learn subject knowledge through another language that is not their first one. Motivation can be referred to as “what moves a person to make certain choices, to engage in action, to expend effort and persist in action” (Dörnyei and Ushioda, 2011, p. 3). It plays a significant role in second language learning as it may trigger language learning and is often the driving force to sustain long-term learning (Ellis, 1997; Ryan and Deci, 2000b; Dörnyei and Ushioda, 2011). It is a key factor affecting learning behavior, attitudes, and achievements (Hengsadeekul et al., 2014). Though there have been abundant studies on motivation in the field of English as a Foreign Language (EFL) contexts, very few studies focus on motivational research in EMI contexts. Hence, attention has been called to Foreign Language Learning (FLL) motivation within EMI contexts (Lasagabaster, 2016; Guo et al., 2018; Macaro et al., 2018; Rose et al., 2020; Xie and Curle, 2022). In an EMI context, not only FLL motivation is relevant but also the motivation to learn language and content in an integrated way. Somers and Llinares (2018) coined the term CLIL motivation, which was first used to research secondary school CLIL students’ language and content learning motivation. The concept of CLIL motivation can well be extended to EMI contexts, where language and content also form a complex integrated aim (Brown and Bradford, 2017). In fact, CLIL and EMI are sometimes used as synonymous terms and remain an ongoing issue which notion to adopt in academic studies (Smit and Dafouz, 2012; Costa and Mastellotto, 2022).

This research study is part of a larger project which investigates EMI practices in three non-linguistic disciplines (International Trade, Film Production, and Project Management) in three Chinese universities. This article will specifically report on pre and post semester students’ motivation questionnaires and students’ post focus group interviews (only the motivation part). The study seeks to explore and compare students’ development of EMI motivation as well as anxiety before and after the one-semester EMI courses. Besides, it aims to compare differences in motivation among the three disciplines studied.

## 2. Literature review

### 2.1. Integrative and instrumental motivation

Gardner and Lambert (1972) proposed Integrative and instrumental motivational orientations, two widely-known motivation types, playing a fundamental role in L2 motivation research. Gardner’s (1985, 2001) socio-educational model found that integrative motives and attitudes toward the learning situation correlated with the learner’s language learning motivation. Integrative motivation is formed by three variables, Integrativeness, good attitudes toward the Learning Situation, and Motivation, and can be referred to as “a complex of attitudinal, goal-directed, and motivational attributes. That is, the integratively motivated individual is one who is motivated to learn the second language, has a desire or willingness to identify with the other language community, and tends to evaluate the learning situation positively” (Gardner, 2001, p. 6). Meanwhile, instrumental orientation reflects “the practical value and advantages of learning a new language” (Gardner and Lambert, 1972, p. 132). Instrumental motivation refers to practical and external factors that stimulate motivation, such as pursuing achievement in a career (Gardner, 2001).

### 2.2. Intrinsic and extrinsic motivation

Deci and Ryan (1985) Self-Determination Theory (SDT) identified different types of human motivation based on the source of motivation, for example, intrinsic and extrinsic motivation, which are highly relevant to second language learning motivation (Lai, 2013). Intrinsic motivation “is the energy source that is central to the active nature of the organism. Its recognition highlighted the important points that not all behaviors are drive-based, nor are they a function of external controls” (Deci and Ryan, 1985, p. 11). In contrast to external drives, intrinsic motivation is human nature and is driven by interest, enjoyment, and satisfaction (Ryan and Deci, 2000b). Equally important is extrinsic motivation. According to Ryan and Deci (2000b, p. 71), the term extrinsic motivation refers to “the performance of an activity in order to attain some separable outcome and, thus, contrasts with intrinsic motivation, which refers to doing an activity for the inherent satisfaction of the activity itself.” This definition differentiates the fundamental distinction between the two types of motivation, which is that intrinsic motivation motivates individuals because of inner feelings such as fun and enjoyment and extrinsic motivation is typically linked with external outcomes, for example, rewards and approval from self or others (Ryan and Deci, 2000a).

### 2.3. CLIL/EMI motivation and anxiety

The motivational concepts reviewed above have mainly been used in FLL motivation studies in which language learning is the mainstream focus. What distinguishes EMI from FLL motivation is the inclusion of content learning together with language learning and their integration. FLL motivation is crucial in CLIL and EMI contexts but Somers and Llinares (2018, p. 5) noted that CLIL motivation does not separate content and language learning, it focuses on integrating the two and treats language and content learning together instead of two isolated concepts. According to Somers and Llinares (2018), two major motives for CLIL are: intrinsic CLIL motivation and instrumental CLIL motivation. Intrinsic CLIL motivation was defined as “the participation in CLIL classes for its inherent satisfaction” (Somers and Llinares, 2018, p. 5). That means CLIL learners will enjoy learning content through a foreign language and feel content intrinsically through the experience. Another motive is instrumental CLIL motivation, which refers to “the usefulness of participating in a CLIL program as a means to achieve an ulterior motive” (Somers and Llinares, 2018, p. 6). This is related to whether the CLIL experience could be beneficial for achieving practical goals, for example, for pursuing a higher degree study or applying for a professional job.

Anxiety is a significant factor to consider in many educational fields including language learning, where previous research shows that anxiety has a great negative impact on second language learning (Horwitz, 2001). Similarly, anxiety may also be present in CLIL classes due to “the threatening aspects related to engaging with and (expressing) understanding of content through a foreign language” (Somers and Llinares, 2018, p. 5). Such anxiety may affect intrinsic CLIL motivation negatively. While the CLIL approach can bring learners enjoyment, it may also increase their anxiety toward learning due to the challenges and difficulties that integrating content and language entails.

CLIL motivation may indeed be relevant in EMI contexts, as EMI is also argued to stimulate students’ motivation (Hellekjær, 2010; Hengsadeekul et al., 2014; Doiz and Lasagabaster, 2018) mainly thanks to its proposed dual benefits in language and content learning outcomes. This study adopts Somers and Llinares’ (2018) concept of CLIL motivation and anxiety and extends it to EMI contexts. EMI motivation would therefore include the intrinsic and instrumental CLIL motives and anxiety proposed by the authors and it would also add extrinsic and integrative types of motivation, which are also highly relevant for EMI learning settings. EMI motivation and anxiety offer a more holistic view of students’ motivation and anxiety in EMI learning settings than just exploring FLL motivation alone. The concept of EMI motivation used in this study does not specifically include Dörnyei’s (2005) and Dörnyei’s (2009) L2 Motivational Self System although it has been used in the previous EMI literature which will be reviewed in the following section.

### 2.4. FLL motivation and anxiety research in EMI contexts

FLL motivation in EMI contexts has been researched far less compared with stakeholders’ attitudes and perceptions on the learning experience. The existing empirical research on FLL motivation offers no consistent consensus but only some shreds of evidence to the focal issue.

Previous EMI studies in Europe generally show that EMI motivates students. Factors such as the ideal L2 self or students’ future career (Hellekjær, 2010; Lasagabaster, 2016; Doiz and Lasagabaster, 2018), and the ought-to L2 self or external pressure (Doiz and Lasagabaster, 2018) appear to play an important role, whereas the L2 community and integrativeness or English-speaking culture show less impact on students’ motivation (Lasagabaster, 2016; Hernández-Nanclares and Jiménez-Muñoz, 2017). Variables such as social status (Lueg and Lueg, 2015) and previous grades (Menéndez et al., 2018) are influential to students’ motivation and some studies suggest that gender as a variable only has minor effects on motivation (Lueg and Lueg, 2015; Lasagabaster, 2016) but others find that female students are more motivated than male students (Menéndez et al., 2018). Students’ L1 is not seen as an important variable in EMI (Lasagabaster, 2016).

It is also important to note that motivation can differ significantly from context to context in relation to cultural, language and socio-economic factors, as motivation is often influenced by the language environment the learner finds him or herself. A second language learning or FLL environment may lead to very different results as regards language motivation (Oxford and Shearin, 1994). Thus, it is vital to take contextual variables into count.

In relation to Asian contexts, inconsistent findings have been found. While some studies concluded that EMI was effective in motivating students, others did not find evidence supporting this claim. Students’ anxiety, however, was detected in many EMI contexts. For example, in Thailand, Hengsadeekul et al. (2014) surveyed 2,252 EMI students, and found out that instrumental motivation ranked first while integrative goals were also positive. Meanwhile, English speech anxiety, fear of negative evaluation, and fear of social comparison had a high but negative correlation with students’ preference for EMI courses. Moving to China, Lei and Hu’s (2014) study showed that students had high anxiety levels in EMI classes and that was negatively associated with their prior English proficiency. Guo et al. (2018) revealed that the EMI group studied obtained exceedingly higher scores in the Extrinsic Goal Orientation factor than the CMI one, thus showing that EMI effectively increased students’ extrinsic motivation. A longitudinal study that assessed engineering EMI students’ changes in perceptions in Turkey revealed that students were motivated because of professional development and language learning benefits. Besides, the findings showed that students’ perception toward content learning through EMI improved through and after the EMI study. They explained that the positive tendency in attitude was detected possibly because the preparatory program they took before entering EMI classes was effective in enhancing their English skills (Sahan and Şahan, 2021). On the other hand, other researchers argued that EMI did not specifically motivate students’ learning or positively affect their content achievement. Wei et al.’s (2017) research study found that students’ English learning motivational intensity was slightly lower than the midpoint score, suggesting they were not strongly motivated to learn English in the EMI context. The researcher noted that the focal university was less privileged, which may have caused the students’ neutral or even negative motivation but students in more prestigious universities might have greater enthusiasm, which indicates that the specific institutional and disciplinary context may also have an impact on students’ motivation. Similar findings by Rose et al. (2020) in Japan and Xie and Curle (2022) in China showed no statistically significant correlation between students’ language learning motivation and academic success, indicating that further research was necessary to generate more robust evidence. Xie and Curle (2022) suggested there should be more longitudinal studies to investigate how motivation develops over time.

As is summarized above, inconsistent findings on the impact of EMI on students’ motivation have been found, thus suggesting more empirical studies are needed. Also, it is necessary to know if students may experience loss or increase of motivation as a result of learning in EMI contexts and causes for the potential changes (Kirkgöz, 2005; Macaro et al., 2018; Xie and Curle, 2022). A single case where students develop their motivation through EMI cannot be generalized to other contexts (Sahan and Şahan, 2021). In fact, students may even get more demotivated, particularly those with limited English proficiency if they face increased greater language-related challenges along the EMI learning process (Kojima, 2021). In addition, it is important to explore motivational differences among disciplines as disciplinary differences in EMI contexts are also crucial to consider (Wei et al., 2017; Macaro et al., 2018; Rose et al., 2020). Rose et al. (2020) noted that institutional and discipline type are also important factors as each EMI context is unique so students’ motivation may vary.

## 3. Methodology

### 3.1. Aim and research questions

The study aims to explore the effects of EMI implementation in China on the students’ learning motivation, more specifically, how students’ EMI motivation and anxiety may change over time and if differences exist among different disciplines. A mixed-methods pre to post research design was used to assess the development of students’ motivation over EMI semesters in three different disciplines: International Trade, Film Production, and Project Management. The following research questions are formulated:

1. How do Chinese university students’ EMI motivation and anxiety toward EMI courses (i.e., International Trade, Film Production and Project Management) develop over the course of a semester?
2. To what extent are students’ EMI motivation and anxiety toward EMI courses different among the three disciplines?

### 3.2. Participants and universities

International Trade (*n* = 96), Film Production (*n* = 45), and Project Management (*n* = 29) students from three EMI courses based on three universities (International Trade course: Shaanxi University of Science & Technology; Film Production course: Xi’an Polytechnic University; Project Management course: Xi’an Eurasia University) in Xi’an, China, participated in the study. Xi’an is located in the center of China and can be classified as a second-tier city. The three courses were all compulsory for students and no pre-selection criteria (i.e., English proficiency test) for students or lecturers were applied. Students accessed university through the National College Entrance Exam (International Trade students: average English score in college entrance exam 115.2, out of 150; Film Production students: average English score in college entrance exam 93.7, out of 150; average English score in college entrance exam 83.8, out of 150). After entering university, they had to take national College English courses to pass College English Tests 4 and 6, which are standardized tests for general English proficiency. Only the International Trade students took two 32-h business English courses before and during the EMI semester. The selection of participants and universities was based on a convenience sampling method; and most importantly, the three EMI teachers gave permission to their classes for data collection. Also, since this study focused on disciplinary differences, we chose those three different EMI courses.

The three courses comprised 32 teaching hours one semester, and only the International Trade students took in total two 32-h business English courses before and during the EMI semester. The International Trade and Film Production teachers were from Spain, and the Project Management teacher was from Croatia, all had rich experience teaching the subject in China. For the International Trade teacher, English was the only instructional language in his classes. For Film Production classes, a Chinese student assistant interpreted the lectures from English to Chinese. More specifically, when the lecturer paused after each subsection, the student assistant explained the content to the class in Chinese, and he also played a role as the communicator between the lecturer and students when their interaction was hindered due to the language barrier. For the Project Management teacher, English was her only instructional language when teaching the course. A Chinese assistant lecturer was interpreting the lectures from English to Chinese and had the same role as in the Film Production class.

### 3.3. Materials and data collection and analysis

Pilot data was collected before the real data collection to refine the design of the instruments. The real data collection took place at the fall semester of 2019. The questionnaires were given to students at the beginning (September) and the end (December) of the semester. The focus group interviews happened only at the end of the semester after the post questionnaire. Written informed consent forms were signed by the participating students and the study was reviewed and approved by the Universitat Autònoma de Barcelona (UAB) Ethics Committee on Animal and Human Experimentation (CEEAH 4728).

#### 3.3.1. Pre and post semester student questionnaires

The pre-post student questionnaires were written in Chinese and included three main question sections, namely biographical information (section 1); perceptions, expectations and attitudes (section 2), developed from previous studies (Galloway et al., 2017; Yang, 2017) on stakeholders’ beliefs and attitudes; and motivation (section 3), adapted from Somers and Llinares’s (2018) work on CLIL motivation. The pre and the post questionnaires had the same content except that the post questionnaire asked whether students had taken extracurricular English courses during the semester in parallel with the EMI courses. Consent information was added at the beginning of the questionnaire.

Data from the questionnaire section two on perceptions, expectations, and attitudes will not be reported in this paper (*cf.* Zhang and Pladevall-Ballester, 2022). This study will only explore two sections from the questionnaires: the first section, including relevant items such as age, gender, major, grade, experience of studying abroad, etc., and the third section, focused on students’ EMI motivation and anxiety, including 18 five-point Likert scale items (q20-37). The items were grouped into five categories, namely intrinsic motivation (q20-22), extrinsic motivation (q23-25), integrative motivation (q26-27), instrumental motivation (q28-31), and anxiety in EMI classrooms (q32-37). The intrinsic motivation category asked about students’ enjoyment learning the subject taught through English and included three items. The extrinsic motivation section elicited information on whether learning the subject through English is importantly associated with pressure from society, parents and peers and it also included three items. Two items were under the Integrative motivation category, focused on whether studying the subject in English would be helpful to know better foreign cultures and meet foreign friends. The instrumental motivation category comprised four items relating to whether studying subjects in English would be beneficial for practical purposes such as studying abroad or finding a job. Six items asked about anxiety in EMI classrooms, based on whether students feel nervous or not while asking or answering questions or speaking in English in EMI classes.

Cronbach’s Alpha was run to assess the reliability of the instrument. The scales were all widely above 0.7 (Intrinsic motivation category: =0.939; integrative motivation category: 0.799; instrumental motivation category: 0.809; anxiety in EMI classrooms: 0.894), except one (extrinsic motivation category: 0.587), which is almost 0.6. Quantitative data was coded (i.e., 1 to 5 from the Likert scale for each participant’s response to each statement) in Excel and then analyzed using SPSS23. Paired t-tests and one-way ANOVA with Bonferroni *post-hoc* tests were adopted to calculate pre to post within-group gains and between group comparisons at pre and post test.

#### 3.3.2. Focus group interviews

The focus group interviews were administered at the end of the semester in Chinese to facilitate communication among students. It took approximately 30 min for each focus group. The data was audio-recorded. Nine questions on students’ attitudes toward the EMI courses, difficulties, benefits, motivation and teaching methodologies were asked. The designed questions were consistent with the topics in the student questionnaires and aimed to collect more in-depth data. However, this study will only report the focus group interview question 6 “What were the motivations for you to take the course (e.g., for further study, work, enjoyment when learning English, etc.)” in relation to motivation and to expand on the quantitative data obtained in the questionnaires. Other themes in the interview (i.e., attitudes and perceptions) are not reported in this study as they are not focusing on motivation. ATLAS. Ti 7.5.7 was used to code and analyze the focus group interview results.

## 4. Results

### 4.1. Development of students’ EMI motivation and anxiety in the EMI courses

The pre-post questionnaire results from the three groups (International Trade, Film Production, and Project Management) will be presented below to address our first research question, which aimed to explore the development of students’ EMI motivation and anxiety in the EMI courses over one semester.

Table 1 shows the data of each group from the pre and post questionnaires and the difference from pre to post data collection times by presenting the mean scores and standard deviations of the five-point Likert scale questions.

**Table 1:** Pre-post student questionnaires (discipline differences).

	International trade (*N* = 96)			Film production (*N* = 45)			Project management (*N* = 29)		
5-point Likert scale	Pre (SD)	Post (SD)	Difference (*p* value)	Pre (SD)	Post (SD)	Difference (*p* value)	Pre (SD)	Post (SD)	Difference (*p* value)
1. Intrinsic motivation	3.63 (0.84)	3.55 (0.87)	−0.08 (*p* = 0.580)	3.23 (0.99)	2.75 (0.81)	**−0.48 (*p* = 0.011)**	3.79 (0.78)	3.31 (0.79)	−0.48 (*p* = 0.070)
2. Extrinsic motivation	3.22 (0.82)	3.37 (0.74)	0.15 (*p* = 0.246)	3.12 (0.69)	3.00 (0.64)	−0.12 (*p* = 0.446)	3.36 (0.68)	3.25 (0.67)	−0.11 (*p* = 0.617)
3. Integrative motivation	4.04 (0.77)	3.89 (0.88)	−0.15 (*p* = 0.345)	3.85 (0.80)	3.55 (0.89)	−0.30 (*p* = 0.108)	3.96 (0.84)	3.70 (0.93)	−0.26 (*p* = 0.190)
4. Instrumental motivation	4.10 (0.61)	3.99 (0.67)	−0.11 (*p* = 0.219)	3.83 (0.78)	3.53 (0.65)	−0.30 (*p* = 0.054)	3.90 (0.76)	3.66 (0.73)	−0.24 (*p* = 0.091)
5. Anxiety in EMI classrooms	3.69 (0.78)	3.27 (0.89)	**−0.42 (*p* = 0.001)**	3.60 (0.87)	3.29 (0.91)	−0.31 (*p* = 0.145)	3.72 (0.93)	3.70 (0.98)	−0.02 (*p* = 0.933)

Bold values are statistical significant.

**Figure 1:**
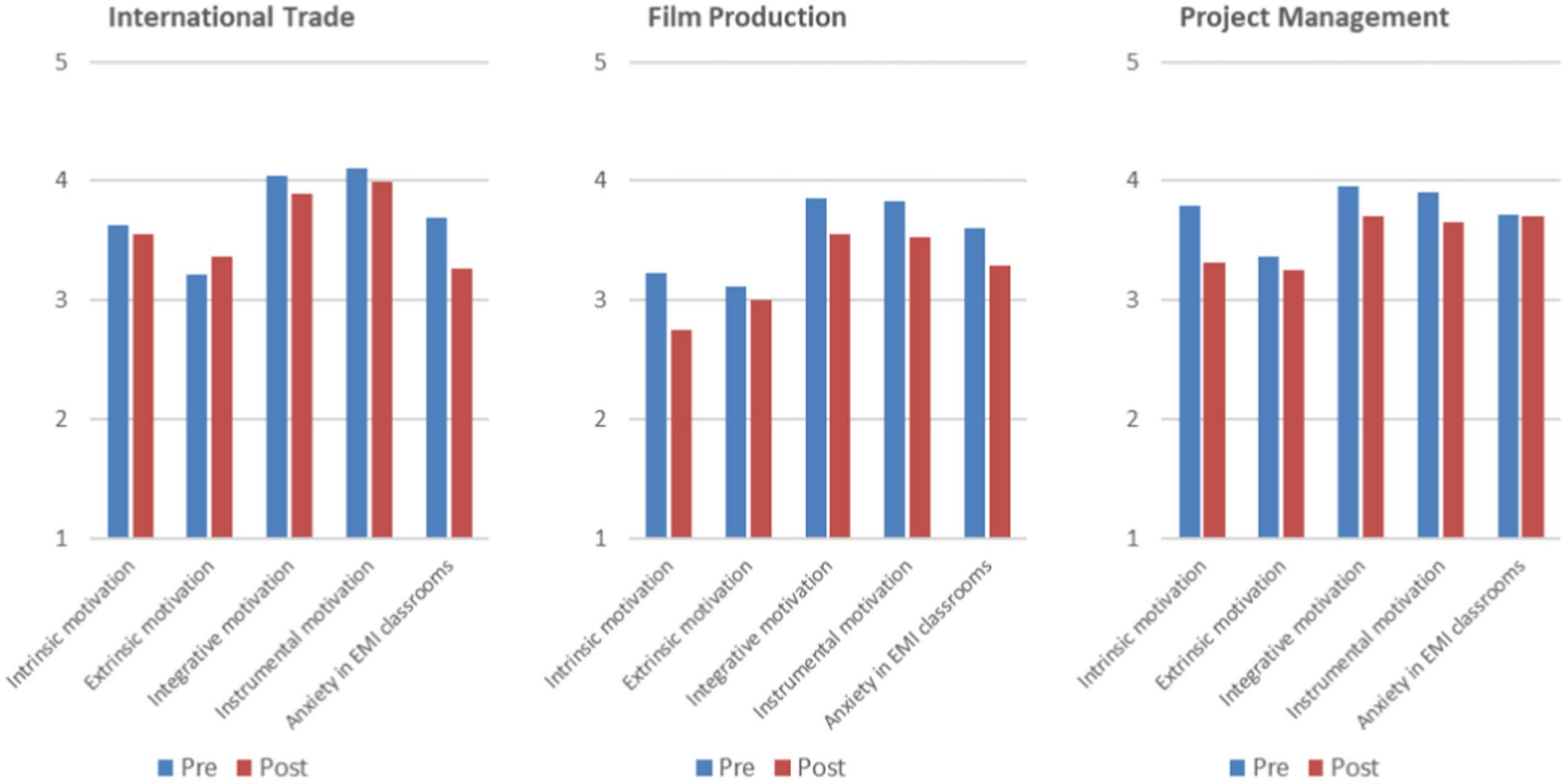
Pre-post student questionnaires (discipline differences).

As can be seen in Table 1 and Figure 1 above, all categories in the three groups decreased their values from pre to post questionnaires, except “Extrinsic motivation” in the International Trade group, where the score slightly increased. Generally, the International Trade group had a smaller decrease in “Intrinsic motivation,” “Integrative motivation,” and “Instrumental motivation” from pre to post questionnaires than the other two groups. Besides, “Anxiety in EMI classrooms” decreased the most in International Trade (0.42), followed by Film Production (0.31), and almost did not change in Project Management (0.02). The Film Production and Project Management groups had the most obvious drop in “Intrinsic Motivation,” with the same value of 0.48. Besides, Film Production scored the lowest among the three groups in the four motivational categories from pre-to-post questionnaires. Project Management had the highest level of “Anxiety in EMI classrooms” at the two data collection times. Importantly, almost all the values were higher than the midpoint 3, considering it is a five-Likert scale questionnaire.

A Wilcoxon signed-ranked test was conducted within each group to test if changes were significant. For the International Trade group, a significant decrease was found in “Anxiety in EMI classrooms”: *Z* = −3.368, *p* = 0.001. For the Film Production group, “Intrinsic motivation” dropped significantly: Z = −2.528, *p* = 0.011. No significant change was detected in the Project Management group. The results indicate that, generally, students’ EMI motivation remained high from pre to post phases, although there was a decreasing tendency. Anxiety was also remarkably high but decreased at the end of the semester.

To understand differences among the motivation variables within the same group at different times, Friedman with paired sample *t*-tests were carried out to compare the means of motivation variables and their differences within each group. Regarding rankings of means among the EMI motivation variables in each disciplinary group, for the trade group and in pre and post tests, “Instrumental motivation” ranked first, followed by “Integrative motivation,” “Intrinsic motivation” and “Extrinsic motivation”; “Anxiety in EMI classrooms” ranked fourth in the pre test and fifth in the post test; for the film group in both pre and post tests, “Integrative motivation” was in the first place, and “Instrumental motivation” in the second place, and they were higher than the other motivation variables; “Intrinsic motivation” was higher than “Extrinsic motivation” in the pre test but their rankings were reversed in the post test. “Anxiety in EMI classrooms” was in the third place in both pre and post tests. For the project group in the two test phases, the variables followed the order of “Integrative motivation,” “Instrumental motivation,” “Intrinsic motivation,” and “Extrinsic motivation”; “Anxiety in EMI classrooms” was in the fourth place in the pre test but ranked first in the post test (see Table 2).

**Table 2:** Pre-post student questionnaires (motivation variable means).

Motivation variable means	Pre trade (Ranking)	Post trade (Ranking)	Pre film (Ranking)	Post film (Ranking)	Pre project (Ranking)	Post project (Ranking)
1. Intrinsic motivation	3.63 (3)	3.55 (3)	3.23 (4)	2.75 (5)	3.79 (3)	3.31 (3)
2. Extrinsic motivation	3.22 (5)	3.37 (4)	3.12 (5)	3.00 (4)	3.36 (5)	3.25 (4)
3. Integrative motivation	4.04 (2)	3.89 (2)	3.85 (1)	3.54 (1)	3.96 (1)	3.67 (1)
4. Instrumental motivation	4.10 (1)	3.99 (1)	3.83 (2)	3.53 (2)	3.90 (2)	3.64 (2)
5. Anxiety in EMI classrooms	3.69 (4)	3.27 (5)	3.60 (3)	3.29 (3)	3.72 (4)	3.67 (1)

As illustrated in Table 3, in the trade group, “Intrinsic motivation” was significantly lower than “Integrative motivation” (pre *p* < 0.001; post *p* = 0.001) and “Instrumental motivation” (pre *p* < 0.001; post *p* < 0.001). “Extrinsic motivation” was also significantly lower than “Integrative motivation” (pre *p* < 0.001; post *p* < 0.001) and “Instrumental motivation” (pre *p* < 0.001; post *p* < 0.001). “Intrinsic motivation” was significantly higher than “Extrinsic motivation” but only in the pre test time (*p* < 0.001). For the film group, “Intrinsic motivation” was significantly lower than “Integrative motivation” (pre *p* < 0.001; post *p* < 0.001) and “Instrumental motivation” (pre *p* < 0.001; post *p* < 0.001). Though “Intrinsic motivation” was significantly higher than “Extrinsic motivation” in the post test (*p* < 0.049), the statistical significance was almost marginal as the *p* value was very close to 0.05. For the project group, “Intrinsic motivation” was significantly lower than “Instrumental motivation” in the post time (*p* = 0.033). “Extrinsic motivation” was significantly lower than “Intrinsic motivation” (pre *p* = 0.009) and “Integrative motivation” (pre *p* < 0.001; post *p* = 0.013) and “Instrumental motivation” (pre *p* = 0.001; post *p* = 0.003).

**Table 3:** Pre-post student questionnaires (motivation variable differences-significance).

Motivation variable (International trade)	Mean difference	Sig. (two-tailed)	Motivation variable (International trade)	Mean difference	Sig. (two-tailed)
Pre intrinsic-extrinsic	0.40	**0.000**	Post intrinsic-extrinsic	0.17	0.088
Pre intrinsic-integrative	−0.42	**0.000**	Post intrinsic-integrative	−0.35	**0.001**
Pre intrinsic-instrumental	−0.48	**0.000**	Post intrinsic-instrumental	−0.45	**0.000**
Pre intrinsic-anxiety	−0.06	0.625	Post intrinsic-anxiety	0.28	**0.057**
Pre extrinsic-integrative	−0.82	**0.000**	Post extrinsic-integrative	−0.52	**0.000**
Pre extrinsic-instrumental	−0.88	**0.000**	Post extrinsic-instrumental	−0.62	**0.000**
Pre extrinsic-anxiety	−0.47	**0.000**	Post extrinsic-anxiety	0.11	0.337
Pre integrative-instrumental	−0.06	0.382	Post integrative-instrumental	−0.10	0.187
Pre integrative-anxiety	0.35	**0.001**	Post integrative-anxiety	0.63	**0.000**
Pre instrumental-anxiety	0.42	**0.000**	Post instrumental-anxiety	0.73	**0.000**
Motivation variable (Film production)	Mean difference	Sig. (two-tailed)	Motivation variable (Film production)	Mean difference	Sig. (two-tailed)
Pre intrinsic-extrinsic	0.11	0.478	Post intrinsic-extrinsic	−0.25	**0.049**
Pre intrinsic-integrative	−0.62	**0.000**	Post intrinsic-integrative	−0.80	**0.000**
Pre intrinsic-instrumental	−0.60	**0.000**	Post intrinsic-instrumental	−0.78	**0.000**
Pre intrinsic-anxiety	−0.37	0.121	Post intrinsic-anxiety	−0.54	**0.009**
Pre extrinsic-integrative	−0.75	**0.000**	Post extrinsic-integrative	−0.54	**0.000**
Pre extrinsic-instrumental	−0.71	**0.000**	Post extrinsic-instrumental	−0.53	**0.000**
Pre extrinsic-anxiety	−0.49	**0.006**	Post extrinsic-anxiety	−0.29	**0.009**
Pre integrative-instrumental	0.03	0.660	Post integrative-instrumental	−0.02	0.883
Pre integrative-anxiety	0.25	0.198	Post integrative-anxiety	0.25	0.173
Pre instrumental-anxiety	0.22	0.246	Post instrumental-anxiety	0.24	0.193
Motivation variable (Project management)	Mean difference	Sig. (two-tailed)	Motivation variable (Project management)	Mean difference	Sig. (two-tailed)
Pre intrinsic-extrinsic	0.44	**0.009**	Post intrinsic-extrinsic	0.06	0.717
Pre intrinsic-integrative	−0.18	0.296	Post intrinsic-integrative	−0.36	0.079
Pre intrinsic-instrumental	−0.12	0.500	Post intrinsic-instrumental	−0.33	**0.033**
Pre intrinsic-anxiety	0.07	0.794	Post intrinsic-anxiety	−0.36	0.102
Pre extrinsic-integrative	−0.60	**0.000**	Post extrinsic-integrative	−0.42	**0.013**
Pre extrinsic-instrumental	−0.53	**0.001**	Post extrinsic-instrumental	−0.39	**0.003**
Pre extrinsic-anxiety	−0.35	0.103	Post extrinsic-anxiety	−0.42	**0.039**
Pre integrative-instrumental	0.06	0.614	Post integrative-instrumental	0.03	0.711
Pre integrative-anxiety	0.24	0.192	Post integrative-anxiety	0.00	1.00
Pre instrumental-anxiety	0.18	0.328	Post instrumental-anxiety	−0.34	0.867

Bold values are statistical significant.

In summary, integrative and instrumental motivations ranked either first or second in all the test phases, indicating that the participating Chinese students had stably higher integrative and instrumental motivation; Intrinsic motivation ranked lower than integrative and instrumental motivation but was averagely higher than extrinsic motivation. Thus, extrinsic motivation was the lowest among the four EMI motivation variables. The four variables throughout the semester were all higher than midpoint 3, except intrinsic motivation in post film test was slightly lower than 3 (2.75). Anxiety in EMI classrooms had been high throughout EMI learning in the semester. Notably, there was no significant difference between integrative and instrumental motivation in any group, implying that the Chinese students had similarly high integrative and instrumental motivation. Generally speaking, the Chinese students had much greater integrative and instrumental motivation than intrinsic and extrinsic motivation, and there existed almost no significant difference between intrinsic and extrinsic motivation either.

### 4.2. Differences in students’ EMI motivation and anxiety among the three disciplines

#### 4.2.1. Quantitative results (pre-post questionnaires)

Our second research question aimed to compare students’ EMI motivation and anxiety toward the EMI courses among three different disciplines over the semester. Pre-post student questionnaire results revealed between-group differences statistically.

One-way ANOVA with Bonferroni *post-hoc* tests were performed to investigate differences among the three groups (Table 4). The results yielded significant differences in all categories except in “Integrative motivation” (*F* = 0.872, *p* = 0.420; *F* = 2.506, *p* = 0.085) and “Anxiety in EMI classrooms” (*F* = 0.216, *p* = 0.806; *F* = 2.334, *p* = 0.100). As for “Intrinsic motivation” (*F* = 4.542, *p* = 0.012; *F* = 13.726, *p* < 0.001), there were significant differences between Film Production and International Trade (*p* = 0.038 in the pre questionnaire; *p* < 0.001 in the post questionnaire) in favor of the latter group, and between Film Production and Project Management (*p* = 0.022 in the pre questionnaire; *p* = 0.017 in the post questionnaire) in favor of the latter group. Regarding “Extrinsic motivation” (*F* = 862, *p* = 0.424; *F* = 4.266, *p* = 0.016), a significant difference was only detected between Film Production and International Trade in the post questionnaire (*p* = 0.012) in favor of the latter group. As for “Instrumental motivation” (*F* = 2.790, *p* = 0.064; *F* = 8.495, *p* < 0.001), International Trade obtained significantly higher scores than Film Production (*p* = 0.001) and Project Management (*p* = 0.040) in the post questionnaire. Thus, the quantitative results found substantial differences in intrinsic motivation, extrinsic motivation and instrumental motivation but not in integrative motivation nor anxiety in EMI classrooms.

**Table 4:** Pre-post student questionnaires (motivation variable differences among the three disciplines).

Motivation variable			Mean difference	Sig. (two-tailed) *p* value	Motivation variable			Mean difference	Sig. (two-tailed) *p* value
Pre intrinsic motivation	Trade	Film	0.40	**0.038**	Post intrinsic motivation	Trade	Film	0.80	**0.000**
		Project	−0.17	1.000			Project	0.24	0.561
	Film	Trade	−0.40	**0.038**		Film	Trade	−0.80	**0.000**
		Project	−0.56	**0.022**			Project	−0.56	**0.017**
	Project	Trade	0.17	1.000		Project	Trade	−0.24	0.561
		Film	0.56	**0.022**			Film	0.56	**0.017**
Pre extrinsic motivation	Trade	Film	0.10	1.000	Post extrinsic motivation	Trade	Film	0.37	**0.012**
		Project	−0.13	1.000			Project	0.12	1.000
	Film	Trade	−0.10	1.000		Film	Trade	−0.37	**0.012**
		Project	−0.24	0.576			Project	−0.25	0.400
	Project	Trade	0.13	1.000		Project	Trade	−0.12	1.000
		Film	0.24	0.576			Film	0.25	0.400
Pre integrative motivation	Trade	Film	0.19	0.570	Post integrative motivation	Trade	Film	0.35	0.097
		Project	0.08	1.000			Project	0.22	0.743
	Film	Trade	−0.19	0.570		Film	Trade	−0.35	0.097
		Project	−0.11	1.000			Project	−0.13	1.000
	Project	Trade	−0.08	1.000		Project	Trade	−0.22	0.743
		Film	0.11	1.000			Film	0.13	1.000
Pre instrumental motivation	Trade	Film	0.28	0.083	Post instrumental motivation	Trade	Film	0.47	**0.001**
		Project	0.20	0.520			Project	0.36	**0.040**
	Film	Trade	−0.28	0.083		Film	Trade	−0.47	**0.001**
		Project	−0.07	1.000			Project	−0.11	1.000
	Project	Trade	−0.20	0.520		Project	Trade	−0.36	**0.040**
		Film	0.07	1.000			Film	0.11	1.000
Pre anxiety in EMI classrooms	Trade	Film	0.08	1.000	Post anxiety in EMI classrooms	Trade	Film	−0.03	1.000
		Project	−0.03	1.000			Project	−0.41	0.107
	Film	Trade	−0.08	1.000		Film	Trade	0.03	1.000
		Project	−0.12	1.000			Project	−0.38	0.241
	Project	Trade	0.03	1.000		Project	Trade	0.41	0.107
		Film	0.12	1.000			Film	0.38	0.241

Bold values are statistical significant.

The tendencies are different in each type of motivation by discipline. The Film Production group scored significantly lower in “Intrinsic motivation” both at the pre and post questionnaires than the other two groups. Also, The Film Production group had a much lower score in “Extrinsic motivation” than the International Trade group in the post questionnaire. Besides, the International Trade group scored significantly higher than Film Production and Project Management in “Instrumental motivation” in the post questionnaire. The significant differences appearing among the three disciplines also imply that the participants from the three universities were different in EMI motivation and anxiety.

#### 4.2.2. Qualitative results (focus group interviews)

Qualitative results from the student focus group interviews at the end of the semester corroborated the quantitative findings. As explained above, this article only reports one question on motivation in the focus group interview, which is “What were the motivations for you to take the course (e.g., for further study, work, enjoyment when learning English, etc.)?” Data from the three groups will be first presented generally and then within each group.

##### 4.2.2.1. Course optionality

All the students pointed out that they took the courses because they were compulsory. Yet, the International Trade and Film Production students reached a consensus that they would have chosen the courses even if they were optional. In contrast, only one student from Project Management stated that she would have attended it even if the course was optional, while others either answered negatively or gave no explicit answer; Such discrepancies across disciplines were likely associated with students’ perceptions toward content learning in the EMI courses. The International Trade and Film Production students held positive attitudes toward content learning in the EMI courses and expressed willingness to take them if they were optional. In contrast, the Project Management students were more reluctant to take an optional EMI course and held a more negative attitude toward content learning in the EMI course. This might be due to the fact that the course content was perceived as not closely related to their field. It seems that the relevance of the course content to students’ fields had some impact on their motivation to take the course.

##### 4.2.2.2. Major motivations for each group

There were two major motivations for the International Trade students. First, they hoped to improve English proficiency by taking the EMI course. This is because their discipline is highly associated with the use of English, and they are required to have a good command of English to be competitive in future development; also, they believe that English as the *lingua franca* is a practical skill in life. Second, they were motivated by the course content as it would be beneficial to their future development. Particularly, they expect to have their previously learned subject knowledge to get consolidated by understanding some concepts deeper. In addition to these major reasons, the students highlighted that they seldom had taken courses taught by international lecturers, thus this course greatly arose their interest. They emphasized their lack of experience taking courses taught by foreign teachers in secondary schools.

*“International Trade Student F: Before entering university, we seldom had foreign lecturers’ courses at junior and senior middle schools. We want to experience such a course out of our own interest. The other reason is that I want to practice oral English as far as I am concerned because if we don’t practice it in real life, then it will be useless.”*

Learning subject knowledge was the most important motivation for the Film Production students. They were interested in learning professional film-making knowledge with the EMI lecturer from an international perspective. The students appreciated the foreign EMI lecturer’s contribution to their subject knowledge learning, especially from a different perspective than local teachers, thanks to the foreign teachers’ different life, educational and working experiences. Also, they were motivated because the content knowledge would be helpful for their future career and personal development. Regarding the experience of language learning as a motivation, only one student mentioned it and believed that progress in English proficiency would be a natural result of taking the EMI course. It seems that improving English skills was not seen as the most urgent demand for these students.

*“Film Production Student E: I am interested in this course as it is relevant to my major and my interest. Also, we need admit that the Hollywood film industry in western countries is more mature, from which Chinese filmmakers should learn. I think that I have learned a lot from the teacher.”*

Regarding the Project Management group, students admitted that the course would help self-development and management, but some complained that the course content was not closely related to their major, so many would not have attended it if it were an optional course. Besides, some students mentioned knowing foreign cultures as a motivation that they enjoyed listening to the foreign teacher share her life experience in another country and getting to know different cultures.

*“Project Management Student C: I liked what she told us about her life in another country and stories, I was interested in those things…this course could be an optional one for us journalism students, and students should be able to choose. You know, my major is journalism, and we have to study Project Management? And the content is not Project Management. I am unhappy with it.”*

## 5. Discussion

### 5.1. Development in students’ motivation

Research question (1) sought to explore the development of students’ EMI motivation and anxiety in the EMI courses over one semester. The findings were drawn from the questionnaires and indicated that students’ overall EMI motivation and anxiety remained high from pre to post phases but generally tended to decrease. This decreasing tendency contradicts a study in Turkey where students were more motivated toward professional and linguistic benefits of EMI through and after taking EMI classes (Sahan and Şahan, 2021). Likely, the preparatory program their students took enhanced their English proficiency, but this is not our case. Our students’ decreased motivation was possibly linked to their reported language difficulties due to limited English proficiency (Kojima, 2021). Additionally, our students might have idealized EMI learning since they had very little relevant experience before entering the courses. More specifically about the changes, intrinsic motivation, integrative motivation, instrumental motivation, and anxiety in EMI classrooms all dropped in scores from pre to post questionnaires. Integrative and instrumental motivations were the most highly valued motivational factors, and the scores were similar, followed by intrinsic motivation in the third place. Extrinsic motivation, however, was the least positively weighted motivational factor. Only in the case of extrinsic motivation and only for the International Trade Group, a slight pre to posttest increase was observed. Our focus group interview results validate the questionnaire findings that the students generally had high EMI motivation. Notably, they expressed that the courses were helpful for their personal and professional development; specifically, they mentioned gains in content knowledge, English skills, and international perspectives. In addition, many said they would like to attend the course even if it was not compulsory.

Overall positive scores in EMI motivation are in line with many previous research studies which have shown that students were greatly motivated within EMI contexts (Hengsadeekul et al., 2014; Lasagabaster, 2016; Hernández-Nanclares and Jiménez-Muñoz, 2017; Guo et al., 2018; Menéndez et al., 2018). However, our findings contrast with the studies by Lei and Hu (2014) and Wei et al.’s (2017) in China, where motivational scores were lower. Our particularly high scores in instrumental motivation corroborate findings by many previous studies (Hengsadeekul et al., 2014; Lasagabaster, 2016; Doiz and Lasagabaster, 2018; Somers and Llinares, 2018 for secondary CLIL students) which revealed that students were greatly driven the pragmatic usefulness of learning English and subject knowledge in English for future education or career development or other relevant purposes. This finding is also in line with our focus group interview results where the most widely mentioned motivational reasons to take the courses were improving English proficiency and enhancing subject knowledge for future development, categorized under instrumental motivation.

As for integrative motivation and its same high scores obtained, it echoes Hengsadeekul et al.’s (2014) findings in Asia but contrasts with some other studies in Europe (Lasagabaster, 2016; Hernández-Nanclares and Jiménez-Muñoz, 2017). Seemingly, students in Asia have more curiosity and interest in English-speaking cultures than those in Europe. In addition, students in Asian countries or China may not have had a rich language-learning context or opportunities to use English outside the classroom (Hu, 2008, cited in Lei and Hu, 2014), thus they may be more eager to practice English with those who speak English. Particularly in the case of the present study, the three institutions analyzed were found in less privileged areas and were second-tier universities, where there might be fewer opportunities for students to meet foreign students and lecturers and to practice their English. The focus group interview results support this, as students expressed interest and needs to study with foreign EMI lecturers and know foreign cultures. Many pointed out that they barely had any course taught by a foreign lecturer before and that they valued having one.

Intrinsic motivation was also high in this study, suggesting that students enjoyed the process of learning content through English as a medium of instruction, although they were not as high as instrumental or integrative motivation. This is in line with Somers and Llinares’ (2018) secondary school CLIL study which found that CLIL students (both High-intensity and Low-intensity CLIL groups) had significantly higher instrumental motivation than intrinsic motivation. Besides, it should be noted that Intrinsic motivation tends to be more restrained after childhood (Ryan and Deci, 2000a) and older or adult students may regard enjoyment or satisfaction in learning as less important than instrumentality. The student focus group interview results of our study also corroborate this, as they mainly expressed the importance of learning English and content knowledge rather than enjoying the learning process. Nevertheless, students showed intrinsic motivation as some mentioned they would have attended the courses even if they were not compulsory.

Extrinsic motivation ranked the lowest in this study, but it was still positive and in line with Guo et al.’s (2018) study in China, which showed that EMI students had significantly higher extrinsic goal orientation than CMI peers. This finding can also be validated by the focus group interview results where many of our students mentioned they enrolled in the courses because of their compulsory nature. Similarly, Doiz and Lasagabaster’s (2018) study in Spain found that students had high ought-to L2 self scores as they were aware of external pressure from their parents’ and society’s opinions on the importance of English. Nevertheless, our findings greatly differ from the ones by Lasagabaster (2016) in Spain, where students’ ought-to L2 self scores were not particularly remarkable. Sociocultural differences might explain this discrepancy. External pressure to meet parental and social expectations is possibly a vital driving force of learning in the Chinese context (Guo et al., 2018), but it may not be the case in western countries. In fact, this is not an uncommon phenomenon in Asian countries. Ethical values emphasize family pride, filial piety and the importance of hard work (Chen, 2012). Chinese parents are typically very strict with their children’s study and push them to perform well in academic life. It is a widely accepted social norm to follow parents’ orders as they are traditionally regarded as authorities; comparatively, western students may face less parental pressure and have more freedom to make decisions for themselves. Also, Asian parents, particularly Chinese parents, may regard their children’s success in academic achievement as their most important goal (Luebbe et al., 2018). Interestingly enough, our students did not mention parental pressure in their group interviews, which might have existed, but students might not have wanted to share it. Possibly, since less privileged universities normally require lower entrance scores than other top ones, we assume that students in less privileged universities may face greater parental pressure as their parents may expect them to perform better academically.

Regarding anxiety in the EMI classroom, results indicate remarkably high levels of anxiety, which confirms the results in other studies (Hengsadeekul et al., 2014; Lei and Hu, 2014). Unsurprisingly, students in our study faced great stress using English in the EMI class as they reported lacking sufficient English proficiency and experience in practicing English before entering the EMI course. China tends to be an EFL and poor language learning context where students have few opportunities to use English outside the classroom (Hu, 2008, cited in Lei and Hu, 2014). In our study, students were anxious as they turned more often to the interpreters’ language help (in the case of Film Production and Project Management) and most of the students did not take the chance to interact with the EMI lecturer in English.

### 5.2. Differences in students’ motivation among the three disciplines

Research question (2) dealt with potential differences among the three disciplines under study regarding students’ EMI motivation and anxiety. Generally speaking, the three disciplines followed similar patterns, as instrumental and integrative motivations ranked similarly high, followed by positive but not so high-ranking intrinsic and extrinsic motivation. In addition, anxiety in the EMI classroom was high for the three disciplines. The fact that the three disciplines generally had similar results regarding motivation might be attributed to their shared social, cultural, and educational contexts. The three EMI courses were conducted based on the same city in China, with the local students coming from the same educational and cultural backgrounds and holding similar social norms. The students in the three disciplines were highly interested in knowing English-speaking cultures. They had neither rich experience with EMI nor opportunities to use English before, and thus they were greatly motivated by integrative reasons.

Though there were no significant differences in integrative motivation and anxiety in EMI classroom among the three disciplines, discrepancies in other motivational factors existed. Specifically, the International Trade and the Project Management groups significantly outperformed the Film Production group in intrinsic motivation at the pre and post phase. As for extrinsic motivation, the International Trade group significantly outperformed the Film Production group at the post phase and in relation to instrumental motivation, the International Trade group scored significantly higher than the other two groups at the post phase. This generally shows that the Trade students had the highest level of motivation among the three disciplines. The Project group remained the middle group in terms of motivational factors, and the Film group ranked the lowest among the three disciplines.

The qualitative findings are in line with and could explain the quantitative data. According to the focus group interview results, only the International Trade students were both enthusiastic about improving their language proficiency and content knowledge through EMI, thus showing interest and motivation on the integration of both content and language, whereas most of the Film Production students only expressed their high expectation on subject knowledge learning but showed less enthusiasm with language learning. Besides, the Project Management students pointed out that the course content was not closely related to their major thus many said they would not have attended if the course were optional. This might explain that the International Trade group had the greatest instrumental motivation and the Project Management group was barely motivated intrinsically. The fact that the International Trade group had the highest English scores in College Entrance Exam (see section 3.2) than the other two groups and that only the International Trade group had taken extra English for Specific Purposes (ESP) courses before and during the EMI course may have contributed to their greater motivation as effective preparatory EMI language courses may help reduce students’ linguistic difficulties and increase their positive experience (Sahan and Şahan, 2021).

Furthermore, a crucial factor that influences students’ motivation surely lies in the type of discipline (Hengsadeekul et al., 2014; Guo et al., 2018; Menéndez et al., 2018). Science majors and social science majors may show different students’ motivations (Guo et al., 2018). Likely, students’ motivation may vary from course to course, depending on each specific course’s characteristics (Menéndez et al., 2018). As is illustrated in Hengsadeekul et al.’s (2014) case, business majors had significantly lower social-cultural goals, namely, integrative motivation, than international business majors. The international business major’s more internationalized nature may attribute a higher level of integrative motivation to students. This might explain that the International Trade group had greater overall motivation than the other more domestic-oriented groups (Film Production and Project Management). In addition, it should be noted that the disciplines were from three universities, which may also have had an impact on students’ EMI motivation and anxiety since each institution has its own strengths in certain disciplines and they may require different entrance scores.

## 6. Conclusion

This study has attempted to shed some light on whether and to what extent students might be motivated in EMI contexts over one semester in China. Our findings suggest that students from the three groups all had generally high EMI motivation, which tended to decrease at the end of the semester. Regarding students’ learning anxiety in the EMI classroom, the level was high both at the pre and posttest phases and also showed a decreasing tendency. The International Trade group generally had the highest motivation, and the Film Production group the lowest one, particularly as regards intrinsic (pre-posttest), extrinsic and instrumental (posttest) types of motivation. No significant between-group differences emerged in relation to integrative motivation and anxiety in EMI classrooms at either of the two data collection points.

A number of specific Implications can be offered to both China and international contexts. In terms of pedagogical strategies for EMI teaching, first, lecturers may offer students more interaction opportunities such as answering questions or doing group activities to use English to interact in class and encourage them to do so as this may help students to become more confident in spoken English and eventually reduce their anxiety in the EMI class. Second, EMI lecturers are supposed to also pay attention to students’ language difficulties and make every effort to facilitate content comprehension.

Regarding implications for institutional policy, it would be advisable to ensure that students reach a certain English threshold before entering EMI courses. Standardized English proficiency tests could be adopted. As is supported by our findings, students’ prior English proficiency had an impact on their motivation in EMI contexts. That is, students of higher English proficiency had the greatest EMI motivation. Besides, providing students with English language or ESP courses, before and after students’ enrolment to the EMI courses is also essential and could help enhance students’ English ability. Moreover, universities may offer more multiple optional EMI courses to students to choose the ones that are most relevant to their major and interests. Otherwise, they are forced to attend but may not have high motivation and thus the quality of learning may be negatively affected. Further empirical studies examining students’ motivation and its relation to English proficiency and academic achievement in EMI contexts are needed.

Limitations of the study should also be acknowledged. First, the study only assessed students’ development in motivation over the course of one semester, too short a period. A longer longitudinal study is needed to trace development more robustly. Further research may include a wider variety of qualitative data to obtain more profound insights. Second, there were differences in the number of participants, and the disciplines were based on different universities and the study adopted a convenient sampling method, thus findings may not be generalizable to other contexts as the sample size was small and was not on the same specific institutional context. Besides, it should not be ignored that the International Trade group taking an extra ESP course might have affected the results. Although this research focused on comparing differences among disciplines, further research could make the best efforts to control other variables in order to make the results more valid. Furthermore, it would also be interesting that further research employs a larger number of participants to examine students’ EMI motivation in the same subject within the same university. Last, empirical studies that examine students’ motivation, English proficiency and academic achievement in EMI contexts are also needed.

Our study has indeed contributed new data on EMI in China. It has offered empirical evidence on students’ development of motivation in EMI contexts and confirms the need to conduct longer longitudinal studies on learning motivation.

## Data availability statement

The original contributions presented in the study are included in the article/Supplementary material, further inquiries can be directed to the corresponding author.

## Ethics statement

The studies involving human participants were reviewed and approved by the Universitat Autònoma de Barcelona (UAB) Ethics Committee on Animal and Human Experimentation (CEEAH 4728). Written informed consent to participate in this study was provided by the participants.

## Author contributions

MZ: conceptualization, data collection, and writing the draft. EP-B: conceptualization and revising the draft. All authors agreed to the submitted version of the manuscript.

## Conflict of interest

The authors declare that the research was conducted in the absence of any commercial or financial relationships that could be construed as a potential conflict of interest.

## Publisher’s note

All claims expressed in this article are solely those of the authors and do not necessarily represent those of their affiliated organizations, or those of the publisher, the editors and the reviewers. Any product that may be evaluated in this article, or claim that may be made by its manufacturer, is not guaranteed or endorsed by the publisher.
