# Supplementary material for: Students’ English-medium instruction motivation in three English-medium instruction courses in China
Source: Front Psychol. 2023 Jan 4;13:1077852. doi: 10.3389/fpsyg.2022.1077852 (PMC9846766; doi:10.3389/fpsyg.2022.1077852)
Supplement: Supplementary file 1 [file Table_1.DOCX]

**Additional Material:**

**Post questionnaire (Section 1. Biographic Information & Section 3. Motivation)**

**1. 1. Basic information**

Gender: ­­­­­­________Age: ________Major: __________________

Grade: ________ Program name: _______________

English score of “Gaokao” (Chinese college entrance examination): ________

I have taken extracurricular English courses during this semester. Yes No

If yes, how many hours______ (Those two questions are only asked in the post survey)

I have studied abroad before. Yes No

I have plans to study abroad in the future. Yes No

**3. EMI motivation & Anxiety in EMI classrooms**

**Intrinsic motivation**

| 20. I enjoyed learning the subject through English. | 1 | 2 | 3 | 4 | 5 |
| --- | --- | --- | --- | --- | --- |
| 21. I enjoyed participating in English in the subject taught through English. | 1 | 2 | 3 | 4 | 5 |
| 22. I enjoyed the classes taught through English. | 1 | 2 | 3 | 4 | 5 |

**Extrinsic motivation**

| 23. I think learning the subject through English is important as society values it. | 1 | 2 | 3 | 4 | 5 |
| --- | --- | --- | --- | --- | --- |
| 24. I think learning the subject through English is important as my parents expect me to do so. | 1 | 2 | 3 | 4 | 5 |
| 25. I hope to have a good performance on this course because it’s important to show to others (such as classmates, parents or employers). | 1 | 2 | 3 | 4 | 5 |

**Integrative motivation**

| 26. I believe that studying the subject in English will help me to understand English people and their lifestyle. | 1 | 2 | 3 | 4 | 5 |
| --- | --- | --- | --- | --- | --- |
| 27. I think using English as a medium of instruction will allow me to have more friends from abroad and to speak with English native speakers from different countries. | 1 | 2 | 3 | 4 | 5 |

**Instrumental motivation**

| 28. I think studying subjects in English will be useful for me because I’ll need it for my future studies. | 1 | 2 | 3 | 4 | 5 |
| --- | --- | --- | --- | --- | --- |
| 29. I think studying subjects in English will be useful for me in ﬁnding a good job. | 1 | 2 | 3 | 4 | 5 |
| 30. I think studying subjects in English will be useful for me because people will respect me more if I speak English well. | 1 | 2 | 3 | 4 | 5 |
| 31. I think studying subjects in English will be useful for me because English is an important language in the world. | 1 | 2 | 3 | 4 | 5 |

**Anxiety in EMI classrooms**

| 32. I felt nervous when I had to speak in my EMI classes. | 1 | 2 | 3 | 4 | 5 |
| --- | --- | --- | --- | --- | --- |
| 33. I worried about making mistakes when I spoke in my EMI classes. | 1 | 2 | 3 | 4 | 5 |
| 34. I feel that the other students speak better English than I do. | 1 | 2 | 3 | 4 | 5 |
| 35. I was afraid to be asked questions in my EMI classes. | 1 | 2 | 3 | 4 | 5 |
| 36. I was afraid to ask questions in my EMI classes. | 1 | 2 | 3 | 4 | 5 |
| 37. I felt nervous when I had to speak in English in group work in my EMI classes. | 1 | 2 | 3 | 4 | 5 |
